# T1 Vertebra Pedicular Osteoid Osteoma: Minimally Invasive Surgical Resection Aided by New Integrated Navigation to 3D Imaging Device

**DOI:** 10.1155/2019/7626454

**Published:** 2019-03-18

**Authors:** M. Prod'homme, G. Cavalié, G. Kerschbaumer, S. Valmary-Degano, M. Boudissa, J. Tonetti

**Affiliations:** ^1^University Hospital of Grenoble-Alps (CHUGA), La Tronche, France; ^2^University Hospital of Geneva (HUG), Geneva, Switzerland; ^3^University of Geneva, Medical Division, Geneva, Switzerland; ^4^University of Grenoble, Medical Division, Grenoble, France

## Abstract

We hereby describe a minimally invasive resection of a T1 pedicular osteoid osteoma next to the vertebral canal. The patient had an 18-month report of painful radiculopathy. We performed the surgery under 3D imaging guidance using navigation with an all-in-one device. Full procedure irradiation was 1.17 mSv for a 181-picture acquisition. Complete operative time incision to closure was 58 minutes. Despite sparing the vertebral stability without any fixation, the tumor resection was well-margined, thanks to the focused guidance. After surgery, the patient had complete relief of his symptoms at the 6-month follow-up. 3D imaging system coupled to navigation made the procedure safe without consuming time. The single Surgivisio® device allows comfortable 3D minimally invasive spine navigation surgery with the ergonomics of a C-arm.

## 1. Introduction

Osteoid osteoma is a common benign bone tumor, ranged as the third, and representing 3% of all bone tumors [1]. The patient, typically aged from 5 to 25 years, presents with pain worse at night and relieved by nonsteroidal anti-inflammatory drugs [2]. Usually localized at the lower extremity, on the femur or the tibia for more than 50% of osteoid osteomas, vertebral localization counts for 10 to 20%. The lumbar column is the most likely localization, and the posterior elements are usually concerned [3]. Complete resection is needed to avoid recurrence [4]. Three-dimensional (3D) imaging-guided resection was first described by Doyle and King [5], with a success rate between 77 and 100%. Percutaneous radiofrequency or minimally invasive resection is the current operative treatment of choice, and open resection should be reserved to recurrent cases [2], which can occur within 17 months after initial resection for 16.3 % of cases [6]. Although its accuracy and safety is widely recognized, radiofrequency ablation should be avoided in challenging cases in the spine [7], especially with a lesion within the proximity of neural or vascular structures.

We describe the first case of osteoid osteoma resection at the upper thoracic spine with minimally invasive approach using a new all-in-one 3D imaging and navigation device named Surgivisio®.

## 2. Case Presentation

A 20-year-old male patient, in otherwise healthy condition, came to our outpatient consultation because of painful right upper thoracic radiculopathy for 18 months without traumatism or any associated medical condition. Pain on visual analogic scale (VAS) was 7-8/10. Patient's pain was relieved by nonsteroidal anti-inflammatory drugs. Clinically, there was neither muscle contracture nor pain triggering at palpation. The pain was located 3 cm from the thoracic midline on the right side, with intercostal irradiation.

**Figure 1:**
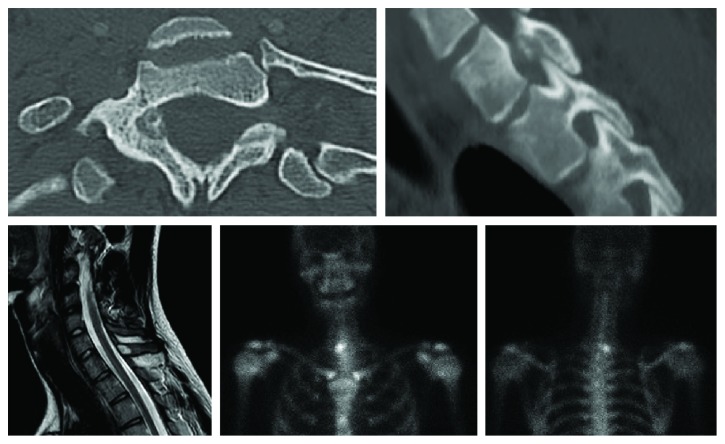
CT scan and MRI of the spine and bone scan. The CT scan shows a rounded osteolytic lesion surrounded by sclerotic bone. Its size is inferior to 15 mm of diameter, within a nidus. MRI shows a T2 hypersignal and a T1 hyposignal on the right side of the T1 vertebra, until the vertebral body with an attachment to the pedicle, and an inflammation of the right T1 root. The bone scan shows hyperfixation of the right T1 vertebra region.

The radiographs showed no osteolysis or deformity. MRI showed a T2 hypersignal and a T1 hyposignal at the medial side of the right T1 vertebra and an inflammation of the right T1 root (Figure 1). The radioisotope bone scanning showed lateral hyperfixation on the right of the T1 vertebra (Figure 1). The CT scan revealed a rounded osteolytic lesion surrounded by sclerotic bone at the medial wall of the right T1 pedicle (Figure 1). The lesion was adjacent to the dura mater and T1 spinal nerve. The aspect was compatible with a nidus, typically found in osteoid osteoma [2].

The decision was made with the patient and our team to perform a resection by posterior approach under 3D imaging guidance. The lesion was abutted to the dura mater, so we could not perform any radiofrequency ablation, which would have required thermal ablation [7]. The surgery should be safe and complete without any risk of further recurrence [4]. We should also preserve the vertebral stability without resorting to screw fixation.

### 2.1. Surgical Technique

**Figure 2:**
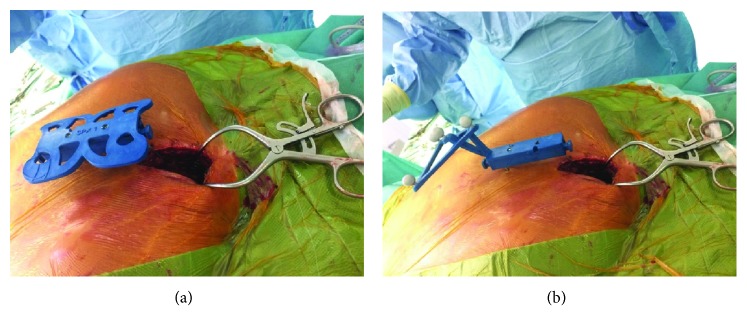
Patient reference in Surgivisio®. The reference frame is fixed to the patient using 2 to 4 pins introduced into spinous processes. We can first put an imaging reference frame (a), then another reference frame for the navigation (b).

**Figure 3:**
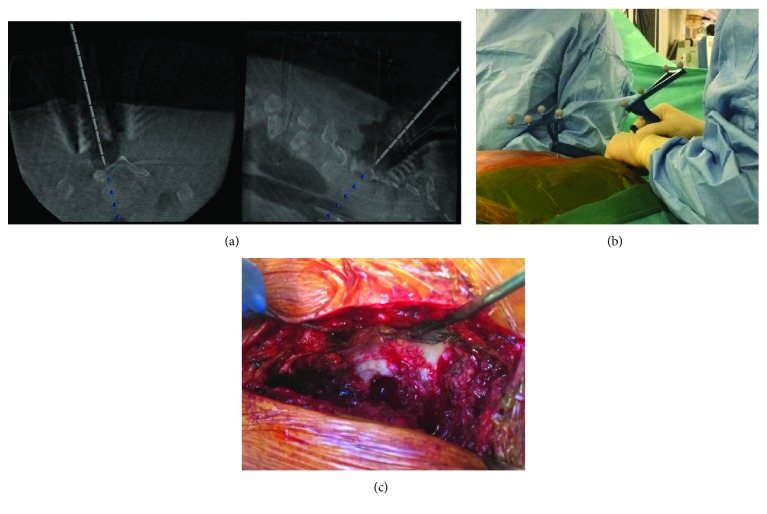
Intraoperative aspect. Navigation imaging (a). We can notice the nidus aspect close to the navigated trocar. The blue landmarks are separated 1 cm each. Reference frame and navigation trocar (b). Mini-open access, with the partial right laminectomy of the T1 vertebra (c) less than 1 cm square-sized. We can notice the pin inside the spinous process of the T1 vertebra.

The patient was in prone position under general anaesthesia. We first controlled the vertebral level with the two-dimensional (2D) mode of our new all-in-one imaging device named Surgivisio® (Surgivisio, Gières, France). The machine is a 2D/3D C-arm made to be combined with surgical navigation. We performed a mini-open posterior approach at the C7-T2 levels (4 cm length). A reference frame was fixed with four pins through the thoracic spinous processes on the midline (Figure 2). We performed a 3D acquisition with 181 images. The surgical team was out of the operating room during the scan. After 40 seconds, the 3D model was available and we could start to navigate. A precalibrated probe was used to palpate the bone surface. We drilled the lower part of the right lamina of T1. A 1 cm square-sized cortical bone surface was removed giving access to the exact localization of the lesion (Figure 3). The tumor was removed using a curette and scraped from the dura mater. Additional resection was made on the surrounding bone using the Kerrison rongeurs. The navigation helped us to control so that the resection looked complete. We closed the wound with drainage for the next 24 postoperative hours. The total operative time (incision to closure) was 58 minutes. The effective dose related to the imaging device was calculated with the PCXMC 2.0 software (PCXMC 2.0, STUK®, Sweden) using the recommendations of the International Commission on Radiological Protection [8]. This result was 1.17 mSv for the whole intraoperative imaging.

### 2.2. Follow-Up

**Figure 4:**
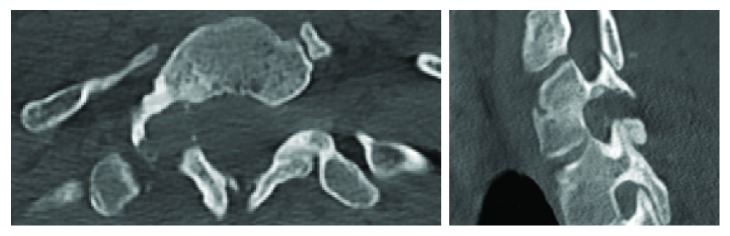
Postoperative spine CT scan after one week. The images confirmed the complete resection of the lesion, without destabilizing the vertebral column.

**Figure 5:**
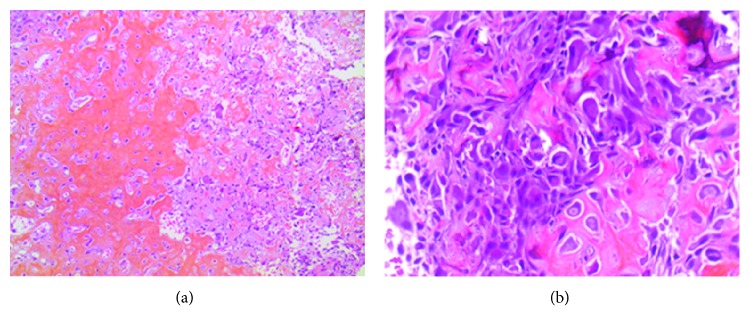
Histopathological examination (standard coloration). An area of hypovascular sclerotic bone surrounding the tumor is seen at low (×100) magnification (a, left part). The intertrabecular space is filled with fibrovascular stroma (a, right part). The central portion of the lesion (nidus) is characterized by differentiating osteoblasts engaged in the production of osteoid tissue. Absence of mitoses and necrosis. (b) ×400 magnification.

During postoperative stay, the patient presented complete pain relief of radiculopathy without any neurological deficit or local infection. His pain was controlled with acetaminophen and tramadol. The second day after surgery, the patient was discharged. CT scan was performed the next week and confirmed full osteoma resection (Figure 4). The histological examination was consistent with an osteoid osteoma (Figure 5). At the 6-month postoperative follow-up, the patient showed no evidence of recurrence. He remained asymptomatic and pain-free (VAS 0/10). He was practicing sports such as bodybuilding and running without any limitation.

## 3. Discussion

Numerous authors recommend percutaneous resection of vertebral osteoid osteomas as a safe and effective procedure [9]. Wang et al. performed a radiofrequency ablation of the osteoid osteomas and osteoblastomas when there is no osteolysis. They reported similar efficacy but higher operative time: a mean of 98 minutes, ranged from 65 to 130 minutes. Campos et al. performed a percutaneous and thoracoscopic video-assisted resection in 60 minutes [10], like our result. Others made curettage or en bloc resection [5]. Regardless of the surgical technique, the objective is to perform a complete resection of the lesion in order to prevent recurrence [2, 4]. In our case, it was impossible to use radiofrequency ablation because of the dura mater abutted to the osteoma.

Several authors also utilized the surgical navigation [10–15] to perform the resection of vertebral osteoid osteomas. Van Royen et al. utilized a preoperative CT scan coupled with surgical navigation and a gamma probe to perform the surgery through a 3-5 cm incision and a mean operative time slightly higher of 84 minutes for 5 patients and reported no recurrence at follow-up [11]. Rajasekaran et al. reported a slightly higher mean operative time of 74 minutes and a similar length of the incision (3-4 cm), with a complete resection in all cases [12]. Kadhim et al. performed a resection under O-arm or C-arm both associated with the navigation [13]. They reported a mean operative time of 123 minutes and a complete resection confirmed intraoperatively by a reacquisition in each case. However, they reported 1 recurrence after 1 year among their 10 patients. Mori et al. reported two successful cases of spinal osteoid osteoma resection through a preoperative CT scan-assisted navigation, without any recurrence after a mean follow-up of 36 months [14]. In 2010, Nagashima et al. published another case of cervical osteoid osteoma resection under CT-guided navigation, without any recurrence after 28 months [15]. All authors concluded a safe and effective technique which allowed for a minimally invasive procedure, thanks to the accuracy of the navigation and the good localization by the CT imaging. All of these previous results are summarized in Table 1.

Literature about irradiation level during vertebral osteoid osteoma resection is relatively scarce. Cheng et al. published a study in 2014 on osteoid osteoma resection under CT guidance [16]. They reported for the axial skeleton a dose length product between 450.20 and 1631.50 mGy·cm, which leads to an effective dose ranging from 7.65 to 31.00 mSv, calculated according to the European Guidelines for Multislice Computed Tomography [17]. These results were clearly higher than ours because the CT scan performs a higher number of images during acquisition than our 3D imaging device. Teeuwisse et al. assessed 31 osteoid osteoma coagulations using CT guidance [18] and reported a mean dose of 2.1 mSv for conventional CT scan use and 0.8 mSv for a scanner with spiral CT fluoroscopy. These results were close to ours. The particular interest in our case was the fact that all operating staff left the operating room during 3D acquisition and received no irradiation at this time. So, the procedure was safe for the patient with minimal irradiation and for the staff additionally.

Besides, the patient had a minimal resection, through the right lamina. This allowed keeping the vertebral stability and to avoid any fixation, as a similar situation in the study of Van Royen et al. [11]. It is well known that a cervical posterior instrumentation is usually not well tolerated [19].

We performed a full resection of an osteoid osteoma at the medial side of the right T1 vertebra pedicle. The precision of the procedure allowed us to respect the vertebral stability without any fusion device. Tridimensional imaging system coupled to navigation enabled a safe, minimal, and time-saving surgery. We experienced a comfortable 3D minimally invasive spine navigation surgery, with the Surgivisio® device, which is combining the ergonomics of a C-arm and an acceptable radiation level.

## Figures and Tables

**Table 1:** Summary of vertebral osteoid osteoma resections using a navigation system.

Study, year	No. of patients	Imaging device	Mean operative time in minutes (range)	Follow-up in months (range)	No. of recurrences
Van Royen, 2004	5	Preop CT scan	84 (70-95)	24.2 (6-33)	0
Rajasekaran, 2008	4	Iso-C 3D navigation	74 (70-90)	24 (20-32)	0
Nagashima, 2010	1	Preop CT scan	*NA*	28	0
Campos, 2012	1	VATS-NAV	60	5	0
Mori, 2016	2	Preop CT scan	*NA*	36 (30-42)	0
Kadhim, 2016	10	3 O-arm, 7 C-arm	123 (70-300)	19 (7.6-28.8)	1
Current case	1	3D C-arm fluoroscopy	58	6	0

Preop: preoperative; VATS-NAV: video-assisted thoracoscopic surgery and navigation; NA: not available.
